# Noncontiguous multi-tiered spinal tuberculosis associated with sternal localization: a case report

**DOI:** 10.1186/s13256-017-1323-2

**Published:** 2017-07-05

**Authors:** Mariam Erraoui, Bouchra Amine, Latifa Tahiri, Imane El Binoune, Jihane Bahha, Najia Hajjaj-Hassouni

**Affiliations:** Faculty of Medicine and Pharmacy, LIRPOS, URAC 30, Rheumatology, Mohammed V University, Rabat, Sale Morocco

**Keywords:** Case report, Multi-tiered spondylodiscitis, Tuberculosis, Sternal localization

## Abstract

**Background:**

Tuberculous spondylodiscitis is a frequent localization of tuberculosis. Multi-tiered involvement and an association with sternal localization are rare.

**Case presentation:**

We report a case of multi-tiered tuberculous spondylodiscitis with sternal localization in an immunocompetent 41-year-old Arab woman who had inflammatory bilateral sciatica L5 and S1 and a history of low back pain caused by a trauma. Radiography, computed tomography, and a vertebral biopsy were useful for diagnosis. She reacted well to anti-bacillary treatment despite the occurrence of multiple paravertebral and subcutaneous abscesses. The medullar magnetic resonance imaging control performed at 4 months, 12 months, and 1 year after the end of treatment showed a favorable evolution.

**Conclusions:**

To avoid the delay of diagnosis, especially in our endemic context, tuberculosis must be evoked usually. This will improve the prognosis of our patients.

## Background

Tuberculous spondylodiscitis (TS), also known as Pott’s disease, was first described in 1779 by Percival Pott [1]. Osteoarticular tuberculosis (TB) occurs in approximately 20% of all extrapulmonary cases, and the spine is involved in 50% of these cases [2]. Primary sternal osteomyelitis caused by *Mycobacterium tuberculosis* is a rare manifestation of extrapulmonary TB. In clinical series, the reported incidence is approximately 1% of all cases of bone and joint TB [3]. We present a case of atypical spinal tuberculosis in a 41-year-old woman with noncontiguous multiple spinal lesions associated with sternal localization.

## Case presentation

**Fig. 1:**
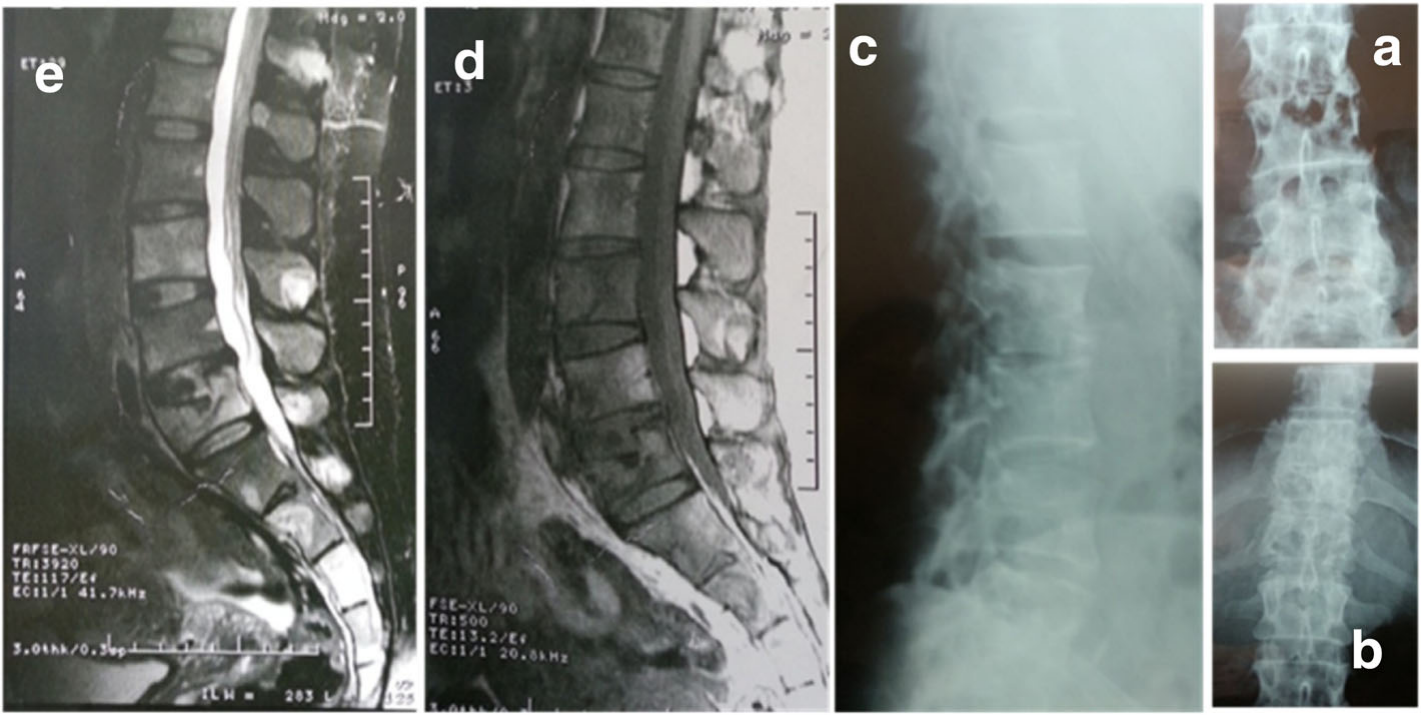
*Right*: radiographs of the lumbar spine taking the last dorsal vertebra showing spinal fractures and disc space narrowing. **a** X-ray of the lumbar spine to face centered on L2 to L3. **b** X-ray of the lumbar spine to face centered on D10 to D11. **c** X-ray of the lumbar spine profile centered on L2 to L3. *Left*: the old lumbar MRI showing L5 fracture with abnormal signal de D11 and D12. **d** T1 sagittal section of lumbar MRI showing L5 fracture. **e** T2 sagittal section of lumbar MRI showing L5 fracture

A 41-year-old Arab woman presented with an inflammatory back pain, associated with bilateral sciatica L5 and S1, 1 year after a minor trauma; she had a history of common low back pain following this trauma. She benefited from a lumbar magnetic resonance imaging (MRI) which objectified a L5 fracture that was related to the trauma (Fig. 1). Thus, she received a symptomatic treatment based on nonsteroidal anti-inflammatory drugs (NSAIDs) and injectable corticosteroid but without any improvement.

At her presentation to our hospital 1 year later, she was bedridden because of pain and she reported weight loss. A physical examination found dorsolumbar spinal syndrome and a neurological examination showed S1 bilateral hyperesthesia in the sensitive territory without motor or genitourinary sphincter deficit.

Laboratory tests found an inflammatory syndrome: erythrocyte sedimentation rate (ESR) was 68 mm in the first hour and C-reactive protein (CRP) was 74 mg/l. A complete blood count (CBC) was normal. Radiography of her dorsolumbar spine (face and profile) showed vertebral fractures D11, D12, and L5 with intervertebral narrowing (Fig. 1).

In this context, a medullar MRI was indicated but because of a health insurance problem, it was delayed. In the bone window, a thoracoabdominal computed tomography scan confirmed vertebral fractures with disc space narrowing and revealed subchondral geodes at D11 to D12, L2 to L3, and L5 vertebrae and paravertebral spine abscess ranged from D11 to L2. In addition, it uncovered mixed lesions of the sternum. Therefore, parenchymal window did not show any abnormality.

A vertebral biopsy directed by scanner with aspiration of the paraspinal abscess was performed. The aspiration product analysis confirmed the diagnosis of tuberculosis by detecting *Mycobacterium tuberculosis* deoxyribonucleic acid (DNA) using polymerase chain reaction (PCR).

A chest X-ray and chest computed tomography (CT) did not show any parenchymal lung lesion or lymphadenopathy. A tuberculin skin test was negative and three sputum acid-fast bacilli stains were negative. Digestive and serological serum tumor markers were also negative.

In order to evaluate the profile of our patient, an immunological assessment was performed. Anti-human immunodeficiency virus (HIV) antibodies, viral hepatitis serology B and C, and syphilis serology tests were all negative.

Anti-mycobacterial therapy was launched with a 2-month initial phase of a combination of four first-line anti-mycobacterial agents (isoniazid, rifampin, pyrazinamide, and ethambutol), followed by a continuation phase of 10 months with isoniazid and rifampin (protocol, 2RHZE/10 RH). Dorsolumbar immobilization was indicated so our patient was given a corset.

During the 4-month treatment, the evolution was clinically characterized by improvement in her back pain, without neurologic or other complications. Her CRP became negative (<6 mg) and her ESR reached a normal value.

**Fig. 2:**
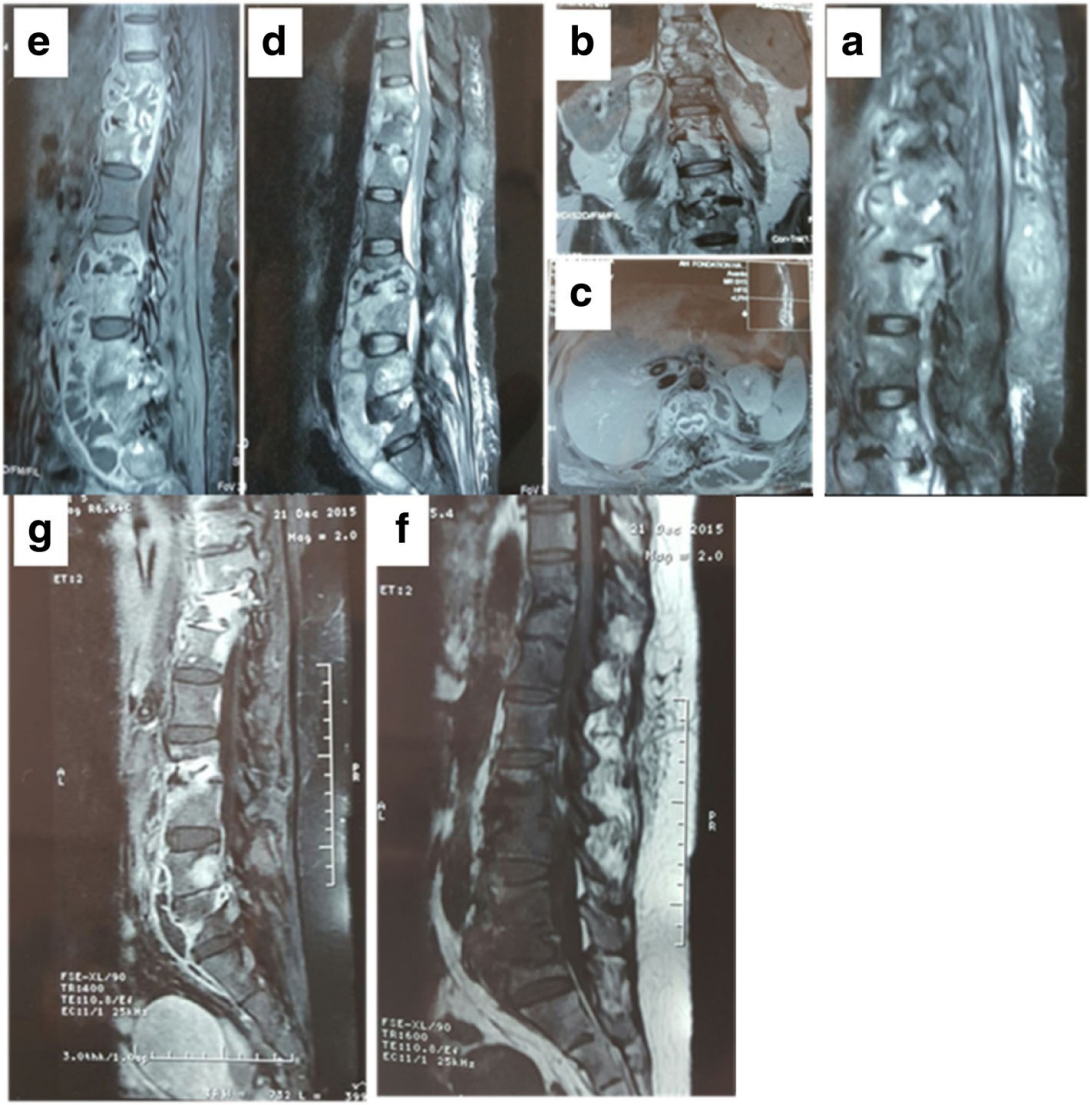
**a**, **b**, **c**, **d** and **e** Dorsolumbar MRI showing the evolution of the destruction and abscess of psoas and subcutaneous. **f** and **g** MRI scan after 12 months of treatment

At the end of this period, she had a painful laterodorsal tumefaction with a rise in CRP and ESR respectively (24 mg/l and 47 mm at the first hour). A medullar MRI revealed more multiple paravertebral abscesses explaining the tumefaction: abscess of her two psoas muscles, subcutaneous dorsal collection with appearance of a new localization at D4 (Fig. 2). Surgical draining of the subcutaneous tumefaction was performed and a drain was set up. Multidrug-resistant tuberculosis was ruled out.

The tumefaction disappeared 15 days later and her CRP was in a normal range. Clinical and biological monitoring was established every week, then every 2 weeks in consultation. Standing up and walking was allowed when she was wearing the corset. The sternal localization was clinically asymptomatic, so we did not need any radiographic control.

After 1 year of treatment, a medullar MRI was performed. It was aimed at finding an improvement in or even a disappearance of some of her injuries. Thus, we stopped the treatment with monthly monitoring. A control of medullar MRI was done 1 year after the end of treatment to find residual paravertebral abscesses with vertebral cicatrization.

## Discussion

Tuberculosis remains the most common cause of death by infectious diseases worldwide [1]. The spinal form is the most common bone joint localization. It occurs at an average age of between 30 and 40 years [1]. The risk factors for this localization are, in particular, the presence of a chronic disease such as diabetes or chronic kidney failure, a long-term corticosteroid therapy, or HIV infection [1, 4]. The concept of trauma that sets off the infection is also reported as a contributing factor in several series, but the pathophysiological mechanism is not well understood. Currently, a precarious factor, such as HIV infection, appears to be an important factor of extended spinal tuberculosis. Nevertheless, occurrence in an immunocompetent patient is not uncommon; several cases are described in the literature. Usually, spine involvement happens through the diffusion of blood from an active primary site (in general, pulmonary site) or a latent site such as lung or lymph node. It can occur by contiguity from a pleural or lymph location. The involvement often concerns two or more adjacent vertebrae. This adjacency is explained by the presence of a single intervertebral artery that supplies two adjacent vertebrae. Noncontiguous multi-tiered spinal involvement is very rare in the literature [5]. It is described especially among children under 7 years due to the persistence of intervertebral disc vascularization [6]. As for adults, a few cases are described but the initial location that explains the multi-tiered involvement is not often found [7]. Cold abscesses are common in spinal tuberculosis (50%). Hence, we should search for them systematically. These abscesses can appear in spite of a well-conducted anti-bacillary treatment, with possible complications namely progression toward the epidural space, the pleural space, the paraspinal muscles, the psoas muscle, and the subcutaneous tissue, and even a dorsal cutaneous fistulization [8]. Surgical draining of these collections is recommended; it accelerates healing and reduces the duration of anti-bacillary chemotherapy [6]. In the case of our patient, the dorsal subcutaneous collection was surgically drained allowing the negativity of CRP and ESR and an improvement in back pain.

Sternal involvement is rare and represents only 1% of all tuberculous osteoarticular damage. In general, it is discovered on the presentation of a sternal pain or swelling or after a sternal fracture. In our case report, the patient had no pain or swelling and the discovery of the sternal involvement was fortuitous following a thoracoabdominal pelvic CT.

Medical treatment is generally effective. Adequate early pharmacological treatment can prevent severe complications. A combination of rifampicin, isoniazid, ethambutol, and pyrazinamide for 2 months followed by combination of rifampicin and isoniazid for a total period of 6, 9, 12, or 18 months is the most frequent protocol used for treatment of spinal TB [9]. The World Health Organization currently recommends a classic quadruple for 2 months (isoniazid, rifampicin, pyrazinamide, and ethambutol) followed by dual combination therapy (rifampicin and isoniazid) for 4 months. The experts’ opinion of the American Thoracic Society, the Centers for Disease Control, and the Infectious Diseases Society of America propose that dual therapy (isoniazid and rifampicin) is continued for 7 months [8, 10]. In our patient’s case, we performed the combination therapy for 10 months due to the multiplicity of abscesses that were inaccessible to surgical draining.

## Conclusions

To avoid the delay of diagnosis, especially in our endemic context, tuberculosis must be evoked usually. This will improve the prognosis of our patients.
